# Increased Agmatine Degradation in Children with Specific Learning Disorder

**DOI:** 10.3390/ijms27073084

**Published:** 2026-03-28

**Authors:** Serkan Kapancık, Elif Abanoz, Serap Çetinkaya, Ahmet Ozan Kaleci

**Affiliations:** 1 Department of Biochemistry, Sivas Cumhuriyet University School of Medicine, 58140 Sivas, Turkey; 2 Department of Child and Adolescent Psychiatry, Sivas Cumhuriyet University School of Medicine, 58140 Sivas, Turkey; 3 Department of Molecular Biology and Genetics, Science Faculty, Sivas Cumhuriyet University, 58140 Sivas, Turkey; 4 Department of Pharmacology, Sivas Cumhuriyet University School of Medicine, 58140 Sivas, Turkey

**Keywords:** specific learning disorder, polyamine, arginine decarboxylase, agmatinase, agmatine

## Abstract

Specific Learning Disability (SLD) describes persistent difficulties in academic skills in reading, writing, and mathematics, despite having normal intelligence. The exact origin of SLD is unknown. However, it is thought that biological factors and environmental conditions, along with genetic factors, contribute to the development of SLD. Agmatine, a neurotransmitter in the brain, plays a role in various biological processes. Agmatine has been reported to mediate antidepressant effects and neuroprotective effects, and it plays critical roles in learning and the processing of learned information into memory. Therefore, this study aimed to determine the relationship between SLD and agmatine metabolism by determining the enzyme levels of arginine decarboxylase (ADC) and agmatinase (AGMAT) in children with SLD. ADC and AGMAT levels in the blood serum of children with SLD and controls were analyzed using ELISA. When ADC levels in children with SLD (30.26 ± 5.06 ng/mL) were compared with those in the control group (29.82 ± 4.95 ng/mL), the difference was not statistically significant (*p* = 0.737). However, AGMAT levels in children with SLD (27.02 ± 4.46 ng/mL) were found to be statistically significantly higher than those in the control group (21.42 ± 3.98 ng/mL) (*p* < 0.001). In light of these findings, we can say that agmatine breakdown is significantly increased in children with SLD.

## 1. Introduction

SLD is a neurodevelopmental disorder that causes persistent difficulties in academic skills such as reading, writing, and mathematics despite normal intelligence levels and is prevalent in society [1,2]. SLD is identified by the DSM-5 diagnostic criteria and, according to these criteria, is seen in different societies and cultures with a high prevalence of 5–15% [3]. The origin of SLD is unknown. However, it is thought that biological factors and environmental conditions, especially genetic factors, contribute to the development of SLD. SLD is diagnosed primarily during school age and is characterized by subgroups such as dyslexia (reading disorder), dysgraphia (writing disorder), or dyscalculia (math disorder). While difficulties in SLD can occur in a single academic skill, difficulties can also occur in multiple academic skills [4].

Therefore, it has been reported that changes in the expression of these three genes may provide insight into the diagnosis and prognosis of SLD [5]. Another study, conducted on blood samples from children with SLD, examined the expression of the MNK1, MNK2, and SYNGAP1 proteins, and the long non-coding RNA *SYNGAP1-AS1*. It has been shown that *MNK1* and *SYNGAP1* gene expression is higher in children with SLD compared to the control group. It has been reported that due to the parallel relationship between SLD and high *MNK1* and *SYNGAP1* gene expression, these genes may play a role in neurodevelopment in the brain [6].

It has been determined that the levels of galectin-1 and galectin-3 proteins in the blood serum are increased in children with SLD. It has been reported that neuroinflammatory mechanisms may be responsible for the pathogenesis of SLD because galectin-1 and galectin-3 proteins play a role in neuroinflammation [7]. When the levels of BDNF, GDNF, NGF, and NT-3 proteins in the blood serum of children with SLD were compared with those in the control group, BDNF, NGF, and NT-3 levels were found to increase dramatically. However, significant differences were reported between the GDNF levels of children with SLD according to SLD severity and reading speed. Based on this, it has been reported that neurotrophins may play a role in the development of SLD and that the increase in BDNF, NGF, and NT-3 protein levels may occur as a response to the SLD process and thus contribute to the protection of neurons [8]. A study investigating the serum levels of MMP-9, TIMP-1, and SIRT-1, which are associated with learning and memory, in children with SLD found that MMP-9 levels decreased, while TIMP-1 levels conversely increased. Based on these results, it has been suggested that MMP-9 may play a role in memory and learning through synaptic plasticity. Furthermore, it has been suggested that changes in the MMP-9/TIMP-1 ratio may contribute to the development of SLD [9]. Decreased ubiquinone levels, an indicator of cognitive performance, were found in the serum of children with SLD. It has been reported that this decrease in ubiquinone levels may be related to the pathogenesis of SLD [10].

Polyamines, organic cations, are widespread in living organisms. They are naturally synthesized in many organisms, from microorganisms to plants and mammals. Furthermore, water-soluble polyamines were first identified in seminal fluids. Subsequent studies have determined that they are synthesized in almost every tissue and cell type. This is primarily because polyamines are vital molecules in cell growth, development, division, and proliferation. Because they play a role in cell division, interruption of their synthesis suppresses cell proliferation and inhibits cell migration [11,12]. Polyamines are synthesized from the amino acid arginine in two ways. In the first, arginine is converted to ornithine by a reaction catalyzed by the enzyme arginase, and from ornithine, they are formed by the enzyme ODC. In addition, agmatine, which belongs to the polyamine group, is formed from the amino acid arginine by the reaction catalyzed by the ADC enzyme, and then another polyamine, putrescine, is synthesized from agmatine by the reaction catalyzed by the AGMAT enzyme. Other polyamines, spermine and spermidine, are synthesized from putrescine [13].

Due to the presence of ADC and AGMAT enzymes in the mammalian brain, agmatine synthesis occurs in the brain, and the agmatine molecule can be stored in neuronal cells. Agmatine, a neurotransmitter in the brain, plays a role in biological processes in the brain through its ability to bind to various NMDA (N-methyl-D-aspartate) and imidazoline receptors [14]. Agmatine has been reported to mediate antidepressant effects in animal models through biological processes via the L-arginine-nitric oxide pathway, NMDA receptors, and α2-adrenoreceptors [15]. Agmatine is also a molecule with neuroprotective effects in the brain. Due to its neuroprotective effects through antioxidant, anti-inflammatory, and anti-apoptotic effects in diseases such as brain injury, stroke, Alzheimer’s, and Parkinson’s, it has been suggested that agmatine may be a therapeutic molecule for these diseases [16]. Additionally, agmatine regulates learning and memory formation through NMDA receptors. NMDA receptors are known to play a central role in synaptic plasticity processes that form the basis of learning and memory capacity in the brain. Learning and memory formation occur as a result of triggering a mechanism called long-term potentiation (LTP) via NMDA receptors, which strengthens synaptic connections. Agmatine modulates the activation of NMDA receptors, thereby modulating learning and memory formation through synaptic connections [17,18]. However, an increase in agmatine levels in the hippocampus during spatial learning in animals has been observed. This has been suggested as evidence that agmatine plays a critical role in learning and in the processing of learned information into memory [19].

In light of this information, it can be assumed that polyamine metabolism, particularly agmatine, may play a significant role in the pathogenesis of SLD, which is characterized by deficits in learning and memory. However, unfortunately, no studies on the relationship between SLD and polyamine metabolism and its enzymes are available in the literature. Therefore, this study aims to illuminate the potential relationship between polyamine metabolism and SLD by examining arginine decarboxylase and agmatinase enzyme levels in children with SLD.

## 2. Results

The sociodemographic and familial characteristics of the participants in this study conducted using the blood serum of 30 pediatric patients diagnosed with SLD and the blood serum of 30 healthy children are compared in Table 1.

According to Table 1, when comparing the sociodemographic characteristics of the children with SLD and the control group, no significant differences were found in terms of age, gender, family income, family type, residence, parental age, or parental education level. The mean age of the children with SLD was 11.60 ± 1.10, while the mean age of the children in the control group was 11.70 ± 1.08, which were quite similar (*p* = 0.961). Gender ratios were similar in both groups, with 46.7% of the children with SLD being boys and 53.3% being girls, while the control group was 53.3% boys and 46.7% girls (*p* = 0.606). Family income levels in the SLD and control groups were mostly above the minimum wage (66.7% and 83.3%, respectively; *p* = 0.136). The SLD and control groups were predominantly nuclear families (90% and 96.7%, respectively; *p* = 0.301). In total, 63.3% of children with SLD and 76.7% of children in the control group lived in rural areas (*p* = 0.260).

The mean age of the mothers of children with SLD was 39.17 ± 3.86, while that of the mothers of children in the control group was 38.40 ± 5.08 (*p* = 0.114). The mean age of the fathers of children with SLD was 42.37 ± 3.85, and the mean age of the fathers of children in the control group was 42.20 ± 3.76 (*p* = 0.978). When the distribution of maternal education levels in the SLD group and the control group was examined, the highest rate in both groups was high school graduation, and the difference between the groups was not statistically significant (*p* = 0.184). The educational levels of the fathers of children in both groups were predominantly primary, secondary, or high school, while the rates of university graduation were low. No statistically significant difference was found between the fathers of children in the SLD group and the control groups (*p* = 0.437). Based on the findings, it can be said that the fact that the groups included in the study were sociodemographically similar minimized the possibility of the results being affected by demographic differences.

**Figure 1:**
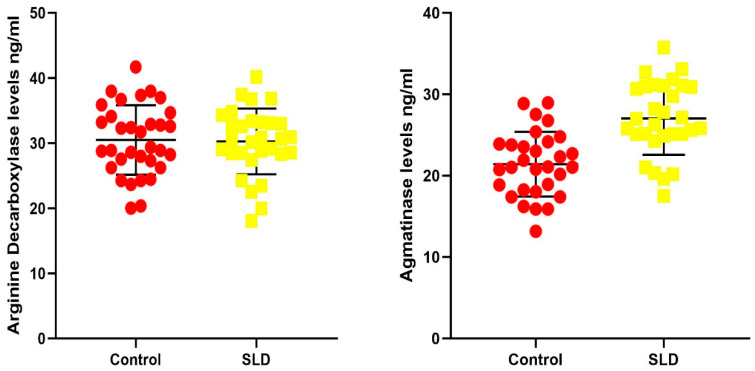
Data on ADC and AGMAT levels in the SLD and control groups. Data are expressed as mean values ± standard deviations.

In the study conducted using the blood serum of 30 children with SLD and the blood serum of 30 healthy children, the ADC and AGMAT enzyme levels of the participants are compared in Table 2 and Figure 1.

Figure 1 compares the ADC and AGMAT protein levels in the serum of children with SLD and those in the control group. Although ADC levels in children with SLD (30.26 ± 5.06 ng/mL) were slightly higher than those in the control group (29.82 ± 4.95 ng/mL), the difference was not statistically significant (*p* = 0.737). However, AGMAT levels were statistically significantly higher in children with SLD (27.02 ± 4.46 ng/mL) compared to those in the control group (21.42 ± 3.98 ng/mL) (*p* < 0.001). The findings suggest that agmatine metabolism is significantly different in children with SLD and that AGMAT levels can be considered a potential biomarker for the diagnosis of SLD.

**Figure 2:**
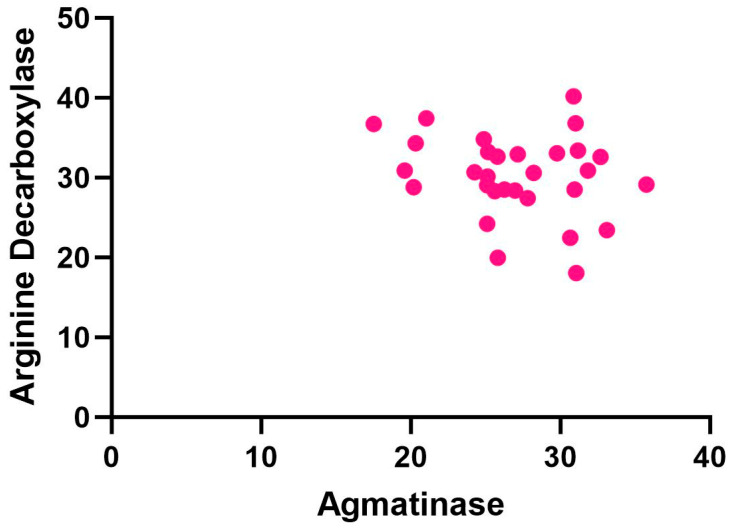
ADC and AGMAT correlation graph.

The correlation levels between ADC and AGMAT levels measured in the blood serum of 30 children with SLD are compared in Figure 2.

Figure 2 shows the ADC and AGMAT correlation graphs in the blood serum of children with SLD. Pearson correlation analysis was conducted to evaluate the linear relationship between ADC and AGMAT. The results provide exploratory findings regarding the correlation between ADC and AGMAT levels, despite the insufficient sample size. According to these preliminary findings, the correlation between ADC and AGMAT levels in children with SLD is not statistically significant, and there is a weak negative relationship between the variables (r = 0.2302, *p* = 0.2211).

## 3. Discussion

The results of the study indicate that, although ADC levels in the serum of children with SLD were normal compared to the control group, AGMAT levels were significantly elevated. This is the first study to demonstrate agmatine metabolism in children with SLD, as no other studies on this topic exist in the literature. In particular, the lack of sociodemographic differences between the children with SLD and the control group suggests that the biochemical difference manifested by elevated AGMAT levels in children with SLD is directly due to SLD.

Although no study has examined polyamine metabolism in SLD, agmatine levels were found to be significantly lower in the plasma of children with autism spectrum disorder compared to healthy controls. Therefore, it has been reported that low agmatine levels may contribute to the development of the disorder and could be considered a target molecule for treatment [20]. Another study investigating arginine, nitric oxide, agmatine, and glutamate levels in children with attention-deficit hyperactivity disorder (ADHD) found increased levels of the amino acid arginine and agmatine in the plasma of children with ADHD. It was suggested that the increased arginine and agmatine levels in children with ADHD may have occurred to inhibit the increased synthesis of nitric oxide and glutamate. The study stated that further studies on the polyamine pathway are needed to understand the pathophysiology of ADHD [21]. In this context, we can conclude that homeostasis in agmatine metabolism may be related to neurodevelopmental and neuropsychiatric conditions.

Agmatine is also known to play a role in learning and memory-related processes. In a study investigating the ameliorative role of agmatine against lipopolysaccharide-induced learning and memory impairment in mice, it was shown that agmatine administration mediated therapeutic effects by preventing memory function losses in mice [22]. In a streptozotocin-induced Alzheimer’s disease rat model, agmatine administration was found to have an ameliorative effect on spatial and emotional memory impairments that occur after streptozotocin exposure. Furthermore, agmatine has been shown to have positive effects against oxidative damage induced by streptozotocin exposure. Based on these data, it has been suggested that agmatine may have therapeutic potential in neurodegenerative diseases, particularly Alzheimer’s disease [23]. It has been shown that the learning and memory impairments induced in another Alzheimer’s disease mouse model induced by beta-amyloid (Aβ1–42) injection can be prevented by agmatine administration to mouse models [24]. In addition, it has been determined that agmatine’s effect in improving memory impairment induced by beta-amyloid (Aβ1–42) in mice is mediated by imidazoline receptors [25]. Although serum agmatine levels were not directly measured in children with SLD in this study, and therefore we cannot definitively speak about the true metabolic flux in agmatine catabolism, an increase in agmatine levels may indicate a decrease in agmatine catabolism. Based on this information, we can predict that disruption of homeostasis in agmatine metabolism, and particularly a decrease in agmatine levels, may contribute to learning disabilities.

## 4. Materials and Methods

### 4.1. Patients and Control Group

The study design was a cross-sectional case–control study. Considering a significance level of α = 0.05 and a desired power of 1 − β = 0.90, a total of 30 participants were included in each group. The post hoc power of is approximately 0.060 for the ADC and approximately 0.993 for the AGMAT. Following clinical evaluations conducted by a child and adolescent psychiatry specialist at the Department of Child and Adolescent Psychiatry, Faculty of Medicine, Sivas Cumhuriyet University, blood samples were taken from 30 children diagnosed with SLD according to DSM-5 diagnostic criteria and 30 healthy children in the control group. The control group consisted of healthy children with similar demographic characteristics to the study group and who did not have any psychiatric disorders or SLD detected during psychiatric evaluation. Without any population limitations for the SLD and control groups, all consecutive participants who presented to the Department of Child and Adolescent Psychiatry, Sivas Cumhuriyet University, between 1 July 2025 and 31 December 2025, and received their first SLD diagnosis were included in this study. No matching was done between the SLD and control groups. Before starting the research, ethical permission was obtained from Sivas Cumhuriyet University Health Sciences Research Ethics Committee dated 12 June 2025 and numbered 2025-06/84. Participants were not taking any medications, including psychotropic drugs. Exclusion criteria for this study included the presence of a comorbid mental disorder and a chronic comorbidity such as type 1 diabetes.

### 4.2. Collecting Blood Samples

Peripheral venous blood samples were collected from the patient and control groups under fasting conditions. After clotting, the samples were centrifuged at 1610× *g* for 10 min to obtain blood serum. Centrifugation was performed at 4 °C, and the resulting serum samples were stored at −80 °C until the day of analysis without undergoing any freeze–thaw cycles.

### 4.3. Analysis of Enzyme Levels in the Polyamine Synthesis Pathway

Protein levels of ADC and AGMAT enzymes in the polyamine synthesis pathway in the serum of children with SLD and children in the control group were analyzed in an ELISA reader using commercial ADC (catalog no: YLA0087HU) and AGMAT (catalog no: YLA0084HU) ELISA kits (YL Biont Lab, Shanghai, China) according to the manufacturer’s instructions. For the ADC ELISA kit, the assay range is 0.2 ng/mL–60 ng/mL, analytical sensitivity is 0.1 ng/mL, intra-assay: CV < 8%, and inter-assay: CV < 10%. For the AGMAT ELISA kit, the assay range is 0.2 ng/mL–60 ng/mL, analytical sensitivity is 0.08 ng/mL, intra-assay: CV < 8%, and inter-assay: CV < 10% [26,27].

### 4.4. Statistical Analysis

The data for our study were loaded into and evaluated using SPSS (Version: 23.0) software. Data normality was evaluated by applying the Shapiro–Wilk test. In Table 1, the chi-square test was used for categorical variables and the Mann–Whitney U test was used for continuous variables to test group differences. In Table 2, the significance test of the difference between two means was used because the assumptions of parametric tests were met. The error level was taken as 0.05.

## 5. Conclusions

In our study, high AGMAT levels along with normal ADC levels indicate accelerated conversion of agmatine to putresine and potentially agmatine deficiency. Based on these findings, we can suggest that disruption of the homeostatic balance in agmatine metabolism may play a role in the pathogenesis of SLD. However, it should be noted that, due to the cross-sectional design of our study, it provides no evidence of a causal relationship. Furthermore, according to the data obtained, AGMAT levels were approximately 25% higher in the SLD group compared to controls (*p* < 0.001); increased AGMAT levels may be a contributing factor to the pathogenesis of SLD, or they may reflect a metabolic response to the underlying neurodevelopmental condition. To reach more definitive conclusions on this matter, these findings need to be biochemically confirmed through repeated studies.

The amino acid arginine is a precursor to the polyamine biosynthesis pathway and is widely obtained through diet. Therefore, individuals’ dietary habits may be among the potential factors affecting the polyamine pathway. One of the limitations of this study is that the detailed dietary habits of the participants were not evaluated. Additionally, in this study, the statistical power for ADC was low due to our sample size being limited to 30 participants. We aim to improve the reliability of these findings by working with larger samples in future research.

## Figures and Tables

**Table 1:** Socio-demographic and familial characteristics of participants.

	SLD	Control	Test	Statistic	df	*p*
Age, years (M ± SD)	11.60 ± 1.10	11.70 ± 1.08	Mann–Whitney U	443.5	--	0.961
Sex, n (%)	Male 14 (46.7), Female 16 (53.3)	Male 16 (53.3), Female 14 (46.7)	χ^2^	0.303	1	0.606
Family income level, n (%) ^†^	Minimum/less: 10 (33.3), Above: 20 (66.7)	Minimum/less: 5 (16.7), Above: 25 (83.3)	χ^2^	2.266	1	0.136
Family type, n (%)	Nuclear 27 (90.0), Extended 3 (10.0)	Nuclear 29 (96.7), Extended 1 (3.3)	χ^2^	1.066	1	0.301
Place of residence, n (%)	Urban 11 (36.7), Rural 19 (63.3)	Urban 7 (23.3), Rural 23 (76.7)	χ^2^	1.276	1	0.260
Mother age, years (M ± SD)	39.17 ± 3.86	38.40 ± 5.08	Mann–Whitney U	434.0	--	0.114
Mother’s education, n (%)	Primary/secondary: 9 (30.0), High school: 16 (53.3), University: 5 (16.7)	Primary/secondary: 16 (53.3), High school: 11 (36.7), University: 3 (10.0)	χ^2^	4.581	2	0.184
Father age, years (M ± SD)	42.37 ± 3.85	42.20 ± 3.76	Mann–Whitney U	441.5	--	0.978
Father’s education, n (%)	Primary/secondary: 13 (43.3), High school: 11 (36.7), University: 6 (20.0)	Primary/secondary: 12 (40.0), High school: 15 (50.0), University: 3 (10.0)	χ^2^	0.561	2	0.437

Note: ^†^ Family income relative to the minimum wage. The statistical significance level is accepted as *p* < 0.05.

**Table 2:** ADC and AGMAT levels in SLD and control groups.

	Control (M ± SD)	SLD (M ± SD)	Test	t	df	*p*
ADC (ng/mL)	29.82 ± 4.95	30.26 ± 5.06	Independent *t*-test	0.34	58	0.737
AGMAT (ng/mL)	21.42 ± 3.98	27.02 ± 4.46	Independent *t*-test	5.14	58	0.001

Note: The statistical significance level was accepted as *p* < 0.05.

## Data Availability

The original contributions presented in this study are included in the article. Further inquiries can be directed to the corresponding author.
